# Role of Information Anxiety and Information Load on Processing of Prescription Drug Information Leaflets

**DOI:** 10.3390/pharmacy5040057

**Published:** 2017-10-16

**Authors:** Shweta S. Bapat, Harshali K. Patel, Sujit S. Sansgiry

**Affiliations:** 1 Pharmaceutical Health Outcomes and Policy, College of Pharmacy, University of Houston, 3455 Cullen Boulevard, 112A Science and Research 2 Bldg, Houston, TX 77204, USA; sbapat3@uh.edu; 2 Amgen, Thousand Oaks, CA 91320, USA; harshali2112@gmail.com

**Keywords:** prescription information leaflet, information processing, information load, information anxiety

## Abstract

In this study, we evaluate the role of information anxiety and information load on the intention to read information from prescription drug information leaflets (PILs). These PILs were developed based on the principals of information load and consumer information processing. This was an experimental prospective repeated measures study conducted in the United States where 360 (62% response rate) university students (>18 years old) participated. Participants were presented with a scenario followed by exposure to the three drug product information sources used to operationalize information load. The three sources were: (i) current practice; (ii) pre-existing one-page text only; and (iii) interventional one-page prototype PILs designed for the study. Information anxiety was measured as anxiety experienced by the individual when encountering information. The outcome variable of intention to read PILs was defined as the likelihood that the patient will read the information provided in the leaflets. A survey questionnaire was used to capture the data and the objectives were analyzed by performing a repeated measures MANOVA using SAS version 9.3. When compared to current practice and one-page text only leaflets, one-page PILs had significantly lower scores on information anxiety (*p <* 0.001) and information load (*p <* 0.001). The intention to read was highest and significantly different (*p <* 0.001) for PILs as compared to current practice or text only leaflets. Information anxiety and information load significantly impacted intention to read (*p <* 0.001). Newly developed PILs increased patient’s intention to read and can help in improving the counseling services provided by pharmacists.

## 1. Introduction

Written prescription information is available through different sources to consumers primarily using prescription drug products. The term “prescription drug labeling” includes the sources such as, container label, patient package inserts (PPIs), medication guides, and consumer medication information (CMI) [1,2,3]. Initially, there was lack of evidence to support the hypothesis that patients would benefit from written information. When PPIs were introduced, pharmacists were of the opinion that this would escalate the cost of prescription drugs and that it was against the conviction that doctors and pharmacists knew what was best for the patient [4]. However, a prospective study of prototype PPIs was conducted for three types of drugs. The study demonstrated that PPIs did not increase the proportion of returned prescriptions, and it did not encourage patients to report more side effects [4]. Thus, overall written information was considered to provide knowledge about drug effects and use, and consumers were aware about the usefulness of drug leaflets as seen in previous research [5,6].

Furthermore, providing this written prescription drug information was deemed appropriate to ensure patient safety since 1968 [6]. In addition, prior research has shown that people living with chronic conditions are more likely to seek written information [7]. It has been demonstrated that patients who received the leaflets knew about the potential side effects of the treatment and were more satisfied with the information that they received [8]. A recent systematic review was conducted to understand the impact of patient information leaflets used during a consultation by pharmacists. The review confirms that patient information leaflets improve patient’s knowledge and satisfaction and, in the short-term, prescription drug information leaflets (PILs) were also shown to improve adherence to treatment [9]. An open randomized controlled trial was conducted in a psychiatric hospital in Kuwait to study the effect of information leaflets and counselling by pharmacists on antidepressant adherence. It was observed that patients receiving a prescription drug information leaflet (PIL) and counselling from a pharmacist were more likely to adhere to their medication regimen as compared to patients receiving no PIL [10]. 

By 1979, the FDA proposed regulations to require educational leaflets to accompany all prescription drugs [11,12]. Past research shows that despite efforts to improve prescription drug leaflets there are several issues with the leaflets in the current form, which may serve as barriers to their usefulness. Specifically, the prescription information leaflets are lengthy, have small fonts, unorganized, and often have difficult terminology [13,14,15]. Efforts to address the issues with prescription drug information have been continuous over a long period of time. In a study conducted in 2013, patients and pharmacists mentioned that the one barrier to comprehension of drug leaflets was the amount of information conveyed on the prescription drug label. Overall, the study noted that the pharmacists were dissatisfied with current prescription labels as they felt the labels contained excess information, were difficult to comprehend and lacked information that patients desired in a user-friendly format [16]. A study published in 2007 demonstrated that, the US leaflets had significant shortcomings with the omission of vital information for the safe and effective use of the medications [17]. Moreover, patients generally receive multiple sources of information when they pick up the prescriptions i.e., PPIs, medication guide, as well as CMI [1]. These are known as the supplementary forms of medication information. These multiple sources of information become redundant and confusing for the patient. 

As part of its ongoing efforts, In February 2009, the FDA risk communication advisory committee (RCAC) recommended that FDA should adopt a single standard document for communicating essential information about prescription drugs, which would replace CMI, PPI, and medication guides [18]. This study implements the FDA’s recommendation by developing and testing a one-page prescription information leaflet with a uniform format, patient-friendly content, and clinically relevant information [19,20]. For the current study, medication information leaflets would mean the supplementary medication leaflets. 

The study uses concepts from the information processing theory [21,22], cognitive load theory [23], the consumer buying process model [24], and the elaboration likelihood model [25]. The information processing theory states that a consumer that actively reads the information would encode it and store it in his memory [21,22]. However, individuals have a limited capacity to process new information. There is only a certain amount of information that a consumer can process at one time point, beyond which information overload occurs, which could affect their comprehension adversely [21]. Furthermore, in 1899, John Sweller developed cognitive load theory (CLT) while studying problem solving [23]. While studying learners as they solved problems, Sweller found that learners often use a problem-solving strategy called means-ends analysis. He suggested problem solving by means-ends analysis requires a relatively large amount of cognitive processing capacity, which may not be devoted to schema construction. The eventual aim of Sweller was to propose that instructional designers should limit cognitive load by designing instructional materials that use principles to facilitate learning [23]. This concept of designing content to reduce cognitive load was well accepted by researchers across different fields and has been used in this study. 

The consumer buying process can be widely divided into three steps informational, attitudinal, and behavioral [24]. For the purpose of this study, the focus is on the informational stage of the model. In this stage, patients encounter the information, which they attempt to read and understand. In the case of prescription medication leaflets the patients are often faced with dense information resulting from multiple sources of leaflets that are often poorly formatted. This information is often inconsistent, incomplete, and at best, difficult for patients to understand [15]. This aspect has been addressed in our study. With reference to the elaboration likelihood model, elaboration is the extent to which a person thinks about the issue-related arguments contained in a message [25]. According to Petty and Cacioppo, there are two distinct routes of persuasion, the central and peripheral. The peripheral route is where persuasion occurs as a result of simple cue without necessitating scrutiny of true merits of information presented. The central route of persuasion is that which results from a person’s careful and thoughtful consideration of true merits of information presented [25]. In the case of Rx drugs, an individual has little volitional control over making purchase decision or adherence. If we consider the prescription information leaflet as a source of (persuasive) communication then the two most important concepts to facilitate comprehension would be: (i) Motivation to process; (ii) Ability to process. The variable “motivated to process”, for the purpose of this study would be operationalized as individual’s involvement. 

Overall, it can be concluded that leaflets are too complex for people to comprehend [14]. Thus, the aim should be to use the suggested theories and models and provide clinically relevant information in a comprehensible format so that the information load is low and the comprehension is high. In addition, prescription drug information leaflets developed in this manner can help pharmacists to counsel patients and answer any questions that they may have [26]. Community pharmacists have regular contact with consumers and can play a key role in helping consumers with medication problems for specific illnesses [27]. Better comprehension of patient leaflets would result in an increase in patient compliance and rational use of medication, achievement of desired health outcomes and consequent decrease of public healthcare costs [28,29]. A study was conducted in 2012 to identify the type and frequency of services provided through community pharmacies in the United Arab Emirates (UAE) [26]. The results of the survey questionnaire given to community pharmacists indicated that only 29% of the pharmacists offered patient information leaflets when counseling patients [26]. Overall, further research is warranted to evaluate and identify the best method to communicate written information to patients and to help community pharmacists in achieving their goal of increasing patient engagement in the services offered [15]. This study can help by contributing to this goal. 

A study was conducted to determine if increasing the scope and depth of information for written prescription drug information results in information overload. The results demonstrated that respondents who received too much depth or too little scope of information were more likely to be confused, doubtful, and overwhelmed [30]. Jacoby and Speller concluded in their study that consumers make poorer decisions with more information. They emphasized the need to study the quantity and organization of the information presented to consumers [31]. Another prospective cohort study investigated the influence of patient information leaflets on patient anxiety and adherence. It was reported in this study that, in some patients, reading the leaflet aroused anxiety and in these patients increased anxiety was associated with decreased adherence [7]. These studies suggest that further research to study the impact of information overload and anxiety on consumer comprehension is needed. 

This study is part of a larger study that examines the impact of cognitive effort and patient involvement on prescription medication comprehension [32]. It revisits the information processing framework [33], and helps to understand how individuals use written information when making decisions about medications. Factors that were included in this research were individual’s cognitive effort [23] and motivation/involvement [25] required to process the information. The established cognitive principles were used to design the one-page prescription drug leaflet (PIL) and to reduce the cognitive effort needed to read and understand the information. Cognitive principles of weeding [34], off-loading [34,35,36], clustering [37], coding [15,34,35,38], and color [36,39] were used in the development of PILs. The second factor of “consumer involvement” is a concept that is borrowed from the motivation theory. Involvement can be viewed as the motivation to process information [40]. Past research has established that involvement enhances recall and recognition [25,32]. Patient involvement can be classified as situational and intrinsic involvement. Situational factors are more easily manipulated and hence considered in this study [32]. 

The objective of this study was to empirically test the impact of information load and information anxiety on intention to read information from prescription drug information leaflets (PILs) at different levels of cognitive effort and involvement. Cognitive effort could occur at three levels: high (current practice leaflets), medium (one-page text only leaflets), and low (PILs), and involvement were manipulated using situational scenarios at two levels, low and high. This research would add to the evidence base related to prescription drug leaflets as an information source [32]. 

**Our hypotheses were as follows:**

H1: Information anxiety will be negatively associated with intention to read
H2: Information load will be negatively associated with intention to read
H3: Information load will be positively associated with information anxiety

## 2. Materials and Methods

### 2.1. Study Design

This study utilized a cross sectional experimental design and was conducted in the Texas Medical Center (TMC) in Houston, Texas. Participants (581) were selected using the convenient sampling method. In the current study, cognitive effort and involvement were the manipulated variables. Cognitive effort was manipulated at three levels: high (current practice leaflets), medium, (one-page text only leaflets), and low (PILs), and involvement was manipulated using situational scenarios at two levels, low and high. Participants received the experimental leaflets and the involvement manipulation in a random order. Data was collected in and around University of Houston. Locations were identified within preselected colleges from within the University campus. At each location, every second participant was approached. If the student declined participation then the immediate next student was approached. Only individuals greater than or equal to 18 years of age and able to read English were included. The study procedures were approved by the Committee for the Protection of Human Subjects at University of Houston. 

### 2.2. Information Processing Variables

The manipulated variables in this study were cognitive effort and involvement. Manipulation for the cognitive effort was measured using 7-point semantic differential scale having anchors colorful/colorless, easy to read/difficult to read, and more effort/less effort. Similarly, manipulation check for patient involvement was measured using 7-point semantic differential scale in terms of involvement, interest and motivation. In addition to the above, the survey instrument also contained questions on demographic information, general health status, health-related training, involvement in health improvement, if they read leaflets received with prescription medications, and health literacy level. Appendix A summarizes the steps for the experimental process and Appendix B describes the survey instrument. 

### 2.3. Cognitive Effort

Cognitive effort was manipulated using principles of cognitive load theory. The newly developed PILs were expected to exert the lowest cognitive effort followed by one-page text only (medium) and current practice leaflets (high). This was pretested in pilot studies. The reduced cognitive effort for PILs was achieved using well known cognitive principles and pretesting PILs to check if manipulations related to cognitive effort are effective. The following cognitive principles were implemented:

1. Off-loading—Provide information through other route i.e., use of pictures or symbols. In PILs, universally accepted symbols were used to indicate important safety information and when not to take the medication. Further, actual picture of the medication was included at right top corner to help patient identify the medication and avoid medication errors [34,35,41].
2. Weeding and Signaling—Unnecessary information was excluded and important safety warnings were highlighted (signaling) [34].
3. Clustering—Like-information was kept together [15].
4. Chunking of information or Segmentation—Information was divided into sections or segments [42].
5. Coding/Naming—Each section was titled to provide context of the information [37,38].
6. Color—The PILs used a colored box to highlight the most important information. Further, a colored image of the medication was included for helping patients to identify the medication [25].

### 2.4. PILs

While developing the PILs the aim was to reduce cognitive effort i.e., the effort an individual would have to exert to read and understand the information. These were developed for three drugs namely, Celebrex^®^ (celecoxib), Ventolin HFA^®^ (albuterol) and Prezista^®^ (Darunavir). Concepts from the cognitive load theory were used to develop the PILS [23].

The well-established OTC drug facts panel format was adopted while developing the PILs [43,44]. With the use of the “Drug Facts” label, the information is more uniform and easier to read and understand [44]. The Drug Facts label uses simple language and an easy-to-read format to help people compare and select OTC medicines and follow dosage instructions. Along with the standardized format, the label uses plain-speaking terms to describe the facts about each OTC drug. For example, “uses” replaces “indications”, while other technical words like “precautions” and “contraindications” have been replaced with more easily understood words and phrases [44]. Similar to the OTC drug facts label, a red box (border thickness = pt 2) was included to represent clinically most important information required by the patients to use the medication appropriately when developing the PILs. The PILs were developed to be printed on a A4 size paper (see Appendix C). A previous study that had developed a one-page leaflet before was used as reference and the same drug products were used in our study when developing the one-page PILs [45]. The content of drug information was used as considered from the FDA approved label and was tested for content validity by three academic researchers and two physicians.

### 2.5. One-Page Text Only

These pre-existing one-page text only leaflets were developed by a group of researchers lead by Catalina Health^TM^ as a quality improvement (QI) initiative [46] with the goal of providing patients clearer medication information when they pick up prescriptions at the pharmacy. These were used as is without any modifications (see Appendix D).

### 2.6. Current Practice

For the three medications in concern for this study, patient leaflets that a patient would receive in the current practice in a pharmacy at that time were collected from four major chain pharmacies, specifically Kroger, Target, Walgreens, and CVS. To adopt a conservative approach, the PILs and text only formats were compared to the current practice information leaflet that has the least amount of information load (number of pages). (See Appendix E and Appendix F)

### 2.7. Development of Involvement Scenarios

Scenarios were developed based on past literature and information provided by practicing pharmacists. In this study, situational scenarios were used to manipulate patient involvement into two levels—high and low. The participants were given a situational scenario before they viewed each information leaflet. Scenarios were printed in black on A4 size white paper, double-spaced with 16-point font size. In the high involvement scenario, the participant was asked to imagine that they had a life-threatening disease and that reading the leaflet is important because the medication has significant side effects. Conversely, in the low involvement scenario, the participant was informed that the prescription they were picking was a refill for a drug that they have been using for a year and never experienced any problems. Further, they had to reach home soon as they are hosting a party. 

### 2.8. Instrument Design

A prevalidated survey instrument was used to measure Information load, Information anxiety, and Intention to read. Overall there were three measured variables for the research model, specifically, information load, information anxiety and intention to read. The role of information anxiety and information load on intention to read information from prescription drug information leaflets (PILs) was evaluated.

- Information load: The load perceived by an individual for a particular information leaflet. Jacoby, Speller and Kohn (1974); developed the scales to measure information load. Information overload was measured using a 5-item 7-point likert scale [31].
- Information anxiety: Stress/anxiety experienced by the individual when encountering the information. Information anxiety was measured using a 3-item 7-point likert scale [7].
- Intention to read: Intention to read was the primary dependent variable and was defined as the likelihood that the patient will read the information. Many studies in past have used validated scales to measure intention. Measurement of intention involves 5, 7, or 10-point semantic differential scales (very likely-not at all likely) or likert (strongly agree-strongly disagree) scales [25,47,48]. The study used a two item 7-point semantic differential scale having anchors “very likely” and “not at all likely”. A higher score indicated a higher intention to read the information.

### 2.9. Data Collection Process

Three different leaflets were evaluated by each participant (current-practice, text-only, and PILS). Each participant was exposed to the involvement scenario three times. The participant thus reviewed either high or low involvement scenario twice during the process. First, the participant read the introduction to the experiment followed by the involvement scenario, and then evaluated the leaflet and responded to the questionnaire. The same process was followed for the other two leaflets. The participants were informed that they would have as much time as they needed to evaluate the leaflet information. However, the time that was taken to complete the questionnaire was recorded. 

Lastly, the participant responded to demographic questions. The order of evaluation of each leaflet by the participant was randomized [32]. After the participant completed the questionnaire the packet was collected. Participants were thanked for their participation and were provided a gift as a token of appreciation. Note that the participants were not informed about the gift before they completed the survey because that would have increased their involvement. 

### 2.10. Statistical Analysis

The SAS 9.3 statistical package (SAS Institute Inc., Cary, NC, USA) was used to analyze the data set at an a priori significance level of 0.05 after the data were coded and validated. Frequency distributions and measures of central tendency and dispersions were used to describe the sample and participant responses on the instrument. Reliability analyses were performed for all of the domains by calculating inter-item correlations along with Cronbach’s alpha. Note, the survey questions were adopted from pre-validated questionnaire used in the past. Hence, formal validity analyses were not performed. However, content validity was tested using expert judgments. 

Given the repeated nature of the experiment, repeated measures analysis (MANOVA—3 × 2 repeated measures multivariate analyses of variance) was used to analyze the impact of cognitive effort (3) and involvement (2) on the measured variables. The impact of information load and information anxiety on the intention to read was investigated. Post hoc analyses were conducted to determine which of the means in a one-way analyses of variance (ANOVA) are significantly different. Within- subject’s ANOVA and paired *t*-test were conducted for manipulation check. Regression analysis was used for studying the effect of extraneous variables. 

## 3. Results

### Characteristics of Survey Participants [32]

Of the 581 participants who were approached for participation, 360 completed the survey. The response rate was 62%. The majority of the participants were female respondents (62.01%) and mean age was 23.62 (±6.04). The sample included a majority of whites (40.526%) followed by hispanics (26.94%), asian (16.67%) and african-americans (8.89%). All respondents indicated that they at least had high school education and a majority had some type of college education (79.67%). Approximately, 3/4th (72.50%) of the sample had taken or were on prescription medications at the time of the survey. Only 16.11% of the respondents had received professional training in health-related field. The survey indicated that the majority of the total number of patients studied (62.50%) only sometimes, rarely, or never read information leaflets only. Only 5 respondents (1%) had taken Celebrex in the past. Approximately 10% respondents had taken Ventolin in the past and no participant had taken Prezista in past.

Reliability was tested for the measured variables of information overload, information anxiety, and the intention to read. Standardized cronbach’s alpha was found to be greater than 0.7 for all of the domains. Hence, the domains were considered reliable to measure the respective constructs. 

For manipulation checks for cognitive effort and involvement, the mean values were compared across the levels using an ANOVA and *t*-test. The results indicated a significant difference in means across the different levels indicating a successful manipulation. All of the leaflets were significantly different from each other in their level of cognitive effort. The current process exerts the highest cognitive effort, whereas the PILs required the lowest cognitive effort.

MANOVA, was used to identify the (overall main effects and interaction) effect of cognitive effort and involvement on the measured variables (Information anxiety and information overload). The analysis demonstrated that effects of cognitive effort, involvement, and interaction effects were significant on the dependent variables (*p* < 0.001). An ANOVA followed by post-hoc Scheffe’s test was thus preformed to test all individual effects. As the interaction between cognitive effort and involvement was found to be significant in MANOVA, it was included in each univariate analysis.

The ANOVA analyses (Table 1) indicated a significant (*p <* 0.05) effect of cognitive effort across information anxiety, overload, and intention. Further, effect of involvement was significant (*p <* 0.05) for information overload and the intention to read variables. The interaction effect of cognitive effort and involvement was significant (*p <* 0.05) only for information overload and information anxiety variables.

The differences in means for information load between the three types of leaflets and between the two involvement scenarios were statistically significant (*p <* 0.0001) (Table 2). Similar results were seen for information anxiety.

The information anxiety and information overload was lowest for PILS. Further, text only was only significantly better than current process leaflets for all three variables (Table 3). We did an analysis to check which leaflets performed better during the high and low involvement situation. PILS were found to be significantly different than the other two leaflets for both high and low involvement situations. The reduction of cognitive effort for all of the three leaflets was more distinct for individuals with high involvement, which can be seen from the low mean values for high involvement as compared to low involvement for the three variables Information overload, anxiety, and Intention to read.

No significant difference seen between Current process and text only at both levels of involvement. The alpha level considered is 0.05. 

With reference to the analyses of the extraneous variables, we ran regression models and included all of the extraneous variables (age, gender, race, and education level, prescription medication use, health-related training, involvement in health improvement, if they read leaflets received with prescription medications, general health status, and health literacy) in the model along with the variables Information anxiety and information overload with intention to read as the dependent variable. Only information overload, information anxiety, and reading leaflets were significant (*p <* 0.05). 

## 4. Discussion

The study results indicate that cognitive effort was positively and significantly associated with the information load and information anxiety encountered by the patient. In other words, if low cognitive effort is required to read and process an information content, then the information load and information anxiety experienced by the patient will also be low. Similar results were seen in another study where it was concluded that higher levels of cognitive effort lead to greater levels of frustration, confusion, and doubtfulness [49]. This should be considered when providing prescription drug information to individuals [49]. 

The study results indicate that the effect of reduction of cognitive effort was more pronounced in individuals who had a high involvement. The interaction effect reveals that for a material having high cognitive effort i.e., the current practice, the involvement was directly proportional to information load i.e., highly involved people will experience a high information load and anxiety because it would be difficult for them to read and understand the complex information as compared to people in the low involvement group who do not care about reading the information at all. However, for the newer one-page formats i.e., with decrease in cognitive effort, involvement was inversely related to information load and anxiety. For newer one-page format, highly involved individuals experienced lower information load and anxiety. The effect was higher in PILs as compared to the text only format. 

These results reinforce the elaboration likelihood model indicating that those patients who are highly involved use central route of elaboration i.e., read and critically evaluated the information [25]. For such individuals, it is important to simplify information and reduce the information load and anxiety. PILs exerted the lowest information load and anxiety. Past research has shown that chunking, segmentation, pictures, and colors have helped individuals in information processing and recall [15,38,39,50].

Psychological symptoms associated with information overload include feeling overwhelmed or lost [51], and anxious, confused, or depressed [52]. It is speculated that the stress caused by information can reduce a person’s information processing productivity and accuracy [53]. Patients are more likely to read and understand written instructions if they contain simplified illustrations instead of just text. Simple illustrations must be tied to simple, understandable words. Good design and the use of pictures or symbols increase patient recall and reinforces behavior modification. However, anxiety increases the degree to which information processing is driven by threat-related stimuli rather than by knowledge and goals [54]. Thus, reducing information anxiety would be desirable. A study conducted in the South African population validated results similar to our study. In this study, medicine information incorporating pictograms was seen as being better comprehended than documents containing text only [55]. 

As seen in previous research, persons with a higher education level (college or university) tend to have more understanding on product label than those with lower level [56]. The study had recruited younger subjects whose age and education levels were similar. However, in our study, education level did not have any significant effect on intention to read the leaflet. The results of this study could be directly applicable to young adult population because the mean age of the sample was approximately 23 (±6.04) years. Further, the study results indicate that more than 70% of the population had taken prescription medications in past reflecting that the respondents were not naïve to the prescription medication process in USA. However, most of the respondents were naïve to the study medication.

This study has several limitations that must be considered. The generalizability of the study findings may be limited as the sample consisted of younger students and not actual consumers of the drugs used in the study. However, one could note that if young adults found the information overloaded, one would expect the elderly, who are typical consumers of prescription medications, to have a more pronounced effect. Furthermore, it is well known that measuring behavior involves multi-dimensional concepts with many known and unknown variables that can affect the process or behavior under investigation. It is beyond the scope of any research to measure all of the known variables that could affect a behavior. There could be other unknown factors that had an effect on information processing of prescription medication leaflets. This study was a field-experiment, where although the intent is to mimic a natural process, but the participant activities and results would be generated in a controlled environment. For example, the participants read the leaflets because this study required them to read the leaflet and answer questions. In everyday practice, probably they may never read leaflets. It is important to validate the study results in the real-world patients. The manipulations i.e., cognitive effort and involvement were manipulated at fixed categorical levels but in reality, they exist as a continuum. The data was collected using a self-administered survey. It may be possible that the respondents’ may have answered questions in a manner that may be viewed favorably by others. Also, the validity of the response at individual level could not be checked. However, emphasis on confidentiality of responses might have controlled this to an extent.

## 5. Conclusions

There is a need to balance the information that is provided to consumers of prescription drugs. The information should be sufficient for decision making, and also such that information overload is avoided when it is referenced by the individuals. Cognitive effort was positively and significantly associated with the information load and information anxiety, while information load and anxiety had a significant impact on the intention to read leaflets. There was a significant difference in information load and information anxiety scores across the three leaflets and two levels of involvement. PILs exerted the lowest information load and anxiety when compared to current practice or text only leaflets. Finally, the results of this study can be used by the FDA to compare different one-page formats and develop the most effective standardized patient-directed prescription information leaflet by reducing the information overload and anxiety for consumers. The development of information leaflets with the appropriate layout and design can help pharmacists to offer useful information leaflets to their patients during counseling.

## Figures and Tables

**Table 1:** Effect of cognitive effort and involvement level on mean scores of Information overload, information anxiety and intention to read.

Variable ^a^	Levels	DF	F Value	*p*-Value
Information overload	*Cognitive effort*	2	261.02	<0.0001
	*Involvement*	1	52.48	<0.0001
	*Cognitive effort × involvement*	2	32.32	<0.0001
Information anxiety	*Cognitive effort*	2	198.95	<0.0001
	*Involvement*	1	14.91	0.0001
	*Cognitive effort × involvement*	2	30.18	<0.0001
Intention to read the leaflet	*Cognitive effort*	2	87.73	<0.0001
	*Involvement*	1	67.63	<0.0001
	*Cognitive effor × involvement*	2	2.45	0.0871

^a^ Information overload was measured using a 5-item, 7-point likert scale; Information anxiety was measured using a 3-item, 7-point likert scale; Intention to read the leaflet was measured using a 2-item, 7-point semantic scale.

**Table 2:** Effect of cognitive effort and involvement level on mean scores of Information overload, information anxiety and intention to read.

Variable	Level	Outcome Variables (Mean ± SD)		
		Information Overload	Information Anxiety	Intention to Read
Involvement	*High*	4.20 (±1.74) ^a^	3.41 (±1.97) ^b^	4.97 (±1.93) ^c^
	*Low*	4.77 (±1.46) ^a^	3.77 (±1.70) ^b^	4.08 (±1.89) ^c^
Cognitive effort	*PILs*	3.58 (±1.44) ^d^	2.64 (±1.55) ^fg^	5.23 (±1.70) ^hi^
	*Text only*	4.16 (±1.42) ^de^	3.26 (±1.70) ^f^	4.79 (±1.83) ^h^
	*Current process*	5.72 (±1.15) ^e^	4.87 (±1.53) ^g^	3.54 (±1.94) ^i^

Abbreviation: SD, standard deviation. Scheffe’s test was conducted to test for significant difference between levels. The alpha level considered is 0.05. ^a^ Significant difference between High and Low involvement for Information overload. ^b^ Significant difference between High and Low involvement for Information anxiety. ^c^ Significant difference between High and Low involvement for Intention to read. ^d^ Significant difference between prescription drug information leaflets (PILS) and text only for information overload. ^e^ Significant difference between text only and current process for information overload. ^f^ Significant difference between PILS and text only for information anxiety. ^g^ Significant difference between PILS and current process for information anxiety. ^h^ Significant difference between PILS and text only for Intention to read. ^i^ Significant difference between PILS and current process for intention to read.

**Table 3:** Effect of cognitive effort on mean scores of Information Overload, Information anxiety, and Intention to read leaflets at high and low involvement levels.

**Information Overload**		
**Cognitive Effort**	**Involvement Level**	
	*High*	*Low*
*PILs*	3.01 (±1.22)	4.28 (±1.54)
*Text only*	3.80 (±1.38)	4.32 (±1.26)
*Current process*	5.76 (±1.19)	5.30 (±1.33)
**Information Anxiety**		
**Cognitive Effort**	**Involvement Level**	
	*High*	*Low*
*PILs*	2.15 (±1.28)	3.33 (±1.50)
*Text only*	3.10 (±1.80)	3.42 (±1.85)
*Current process*	5.11 (±1.55)	4.29 (±1.52)
**Intention to Read Leaflets**		
**Cognitive Effort**	**Involvement Level**	
	*High*	*Low*
*PILs*	5.74 (±1.38)	4.72 (±1.83)
*Text only*	5.33 (±1.67)	4.24 (±1.82)
*Current process*	3.82 (±2.12)	3.26 (±1.71)

Abbreviation: SD, standard deviation. Scheffe’s test was conducted to test for significant difference between levels.

## Appendix A. Experiment Procedure for Each Patient

**Steps**	**Description of Each Step**
(1) Initial Subject Participation	Approach an individual and request participation in the study by reciting the informed consent communiqué. After confirming consent, the participant was given a folder
(2) Experimental Process Description	Participant was explained the process and instructed to proceed with the experiment
(3) Experimental process	- Participant will be instructed to read the scenario (involvement scenario) - When Participant indicated that they are ready, they would turn the page and view 1st format (no set time limit) - Participant will then turn the page and fill the questionnaire - The above steps will be repeated for other two formats
(4) Appreciation and gift	Participants will be thanked for their participation and given a token of appreciation
(5) Coding	Questionnaires will be removed from the folder and coded

## Appendix B. Survey Instrument

Prescription Drug Information Leaflet Questionnaire

Based on the information read, responses were then collected from the participants on the below mentioned measures and using a 7-point semantic differential scale as seen below.

**Strongly Disagree**	**Disagree**	**Somewhat Disagree**	**Neutral**	**Somewhat Agree**	**Agree**	**Strongly Agree**
(SD)	(D)	(SoD)	(N)	(SoA)	(A)	(SA)
**1**	**2**	**3**	**4**	**5**	**6**	**7**

Information overload was measured using a 5-item, 7-point likert scale.

Information anxiety was measured using a 3-item, 7-point likert scale.

Please indicate what was your reaction when you tried to read and understand the information leaflet you just viewed:

- There is so much information that I have trouble considering what is important
- I feel confused by the amount of information provided
- I feel the leaflet contains too little information
- It is hard for me to concentrate because of all the information in the leaflet
- The leaflet has so much information to read that it is difficult to remember
- I feel overwhelmed by the amount of information to be understood
- I feel stressed by the amount of information to be understood
- I feel numb and incapable of action by the amount of information to be understood.

A. Intention to read the prescription drug information leaflets was measured using a 2-item, 7-point semantic scale having anchors “very likely” and “not at all likely” as seen below.

Assuming that information would be provided in the format you last viewed, how likely is it that you would read the prescription drug leaflet?

Not at all likely	1	2	3	4	5	6	7	Very Likely

Assuming that information would be provided in the format you last viewed, how likely is it that you would keep the leaflet for future reference?

Not at all likely	1	2	3	4	5	6	7	Very Likely

B. Manipulation check for Cognitive effort was measured using 7-point semantic differential scale having anchors:

Colorful	1	2	3	4	5	6	7	Colorless
Easy to Read	1	2	3	4	5	6	7	Difficult to read
High mental effort required	1	2	3	4	5	6	7	Low mental effort required
Simple Language	1	2	3	4	5	6	7	Difficult language

C. Manipulation check for patient Involvement was measured using a using 7-point semantic differential scale having anchors:

Not at all involved	1	2	3	4	5	6	7	Very Involved
Not at all interested	1	2	3	4	5	6	7	Very Interested
Very Motivated	1	2	3	4	5	6	7	Not at all Motivated
